# The Chemopreventive Activity of Indonesia Medicinal Plants Targeting on Hallmarks of Cancer

**DOI:** 10.15171/apb.2019.025

**Published:** 2019-06-01

**Authors:** Edy Meiyanto, Yonika Arum Larasati

**Affiliations:** ^1^Cancer Chemoprevention Research Center, Faculty of Pharmacy, Universitas Gadjah Mada, Sekip Utara, Yogyakarta 55281, Indonesia.; ^2^Department of Pharmaceutical Chemistry, Faculty of Pharmacy, Universitas Gadjah Mada, Sekip Utara, Yogyakarta 55281, Indonesia.

**Keywords:** Cancer, Chemoprevention, Indonesia, Medicinal plants

## Abstract

Cancer remains a complex disease with increasing global mortality and morbidity. Numerous theories have been established to understand the biological mechanism underlying cancer. One of the most renowned frameworks is the hallmark of cancer proposed by Hanahan and Weinberg that covers ten eminent characteristics of cancer: (*i*) genome instability and mutation, (*ii*) sustaining proliferative signaling, (*iii*) evading growth suppressor, (*iv*) enabling replicative immortality, (*v*) resisting cell death, (*vi*) inducing angiogenesis, (*vii*) activating invasion and metastasis, (*viii*) avoiding immune destruction, (*ix*) tumor-promoting inflammation, and (*x*) deregulating cellular energetics. These hallmarks provide a rational approach to design an anticancer therapy. In the current review, we summarized specific target molecules on each hallmark of cancer. Further, we evaluated the biological activity of several Indonesia medicinal plants against those specific targets. We explicated the anticancer and chemopreventive activities of some medicinal plants that have been used for centuries by local communities in Indonesia, including *Curcuma genus, Brucea javanica, Boesenbergia pandurata, Caesalpinia sappan,* and *Nigella sativa*. Interestingly, these medicinal plants target several hallmarks of cancer, and even *Curcuma genus* exhibited biological activities that target all hallmarks of cancer. Further, we also discuss several strategies to develop those medicinal plants and/or their active compounds as anticancer and chemopreventive agents.

## Introduction

Despite current advancement in cancer prevention, treatment, and diagnostic; cancer remains global jeopardy with 70% of cancer-related deaths take place in low- and middle-income countries.^1^ Characterized by uncontrolled cells proliferation, cancer arises from a genetically transformed cell in a process called carcinogenesis that includes initiation, promotion, and progression stages.^2^ Moreover, cancer holds diversities for each cancer type with particular molecular mechanisms. A notable work of Hanahan and Weinberg simplified the complexity of cancer into ten properties called ‘the hallmarks of cancer’.^3^ The long-term carcinogenesis, together with complex molecular characteristics, makes cancer difficult to be cured completely. Therefore, we need a systematic strategy to eradicate cancer based on its specific molecular markers.

**Figure 1:**
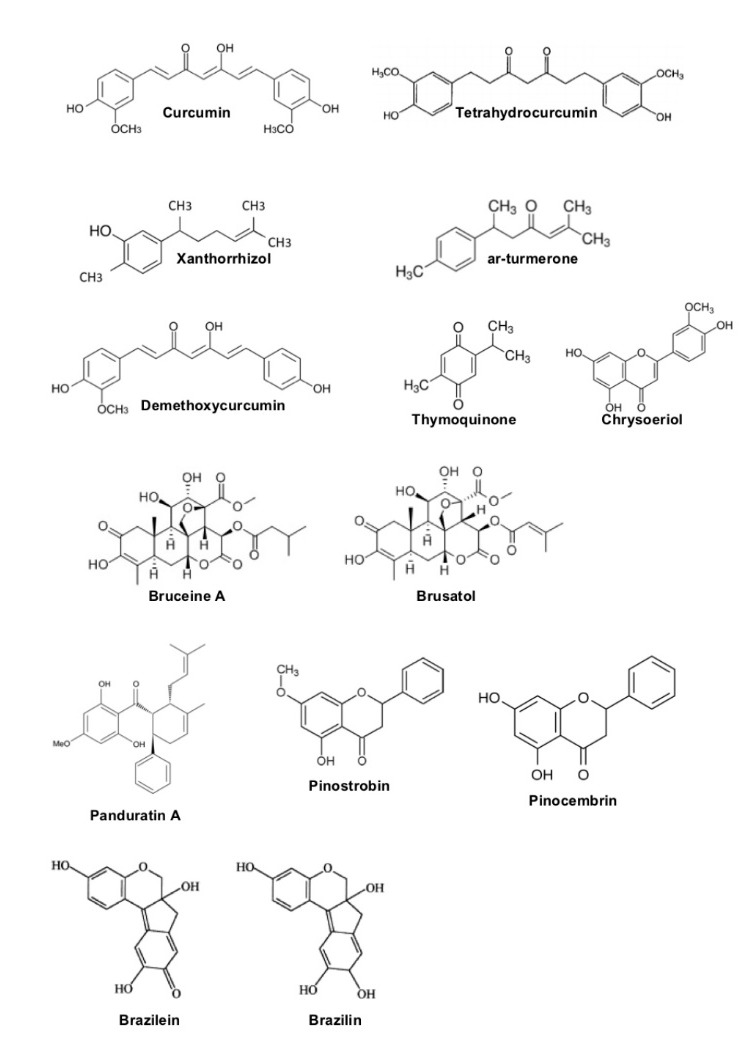
**The structure of various natural products exerting chemopreventive effect.** The structures of curcumin, tetrahydrocurcumin, xanthorrizol, ar-turmerone, and demethoxycurcumin were adapted from Itokawa et al.^5^ The structures of bruceine A and brusatol were adapted from Hall et al.^6^ The structure of thymoquinone was adapted from Bassha et al.^7^ The structures of panduratin A, pinostrobin, and pinocembrin were adapted from Kiat et al.^8^ The structures of brazilein and brazilin were adapted from Laksmiani et al.^9^

The long timeframe between cancer initiation and invasive cancer could be exploited to prevent cancer through cancer chemoprevention, an effort to prevents, inhibits, and/or reverses cancer development.^4^ For this purpose, various natural products show the biological activity to inhibit cancer progression at certain stages with specific molecular targets (Figure 1). Even more, there are many natural substances performing pleiotropic effects in cancer signaling that may share in some stages of cancer progression, i.e. curcumin and thymoquinone.

Indonesia, a mega-biodiversity country, holds potency to provide chemopreventive agents based on its medicinal plants. Herbal medicine is popular there with around 6000 plant species have been used by Indonesian community for various disease prevention and treatment.^10^ In this review, we aim to elaborate the potency of medicinal plants widely used in Indonesia as the chemopreventive agents targeted on the hallmarks of cancer. We would focus on some medicinal plants that have been used for centuries in Indonesia, including *Curcuma* genus, *Brucea javanica*, *Boesenbergia pandurata*, *Caesalpinia sappan*, and *Nigella sativa*.

## Hallmarks of cancer and representation of the Indonesia medicinal plants with specific anti-cancer target

### Genome instability and mutation

Cancer begins with unrepaired mutation(s) in the cells, which are accumulated during the time and disrupt normal cell function. In normal cells, various DNA repair pathways rectify almost all DNA mutations, i.e. non-homologues end-joining (NHEJ) and homologous recombination (HR).^11^ Interestingly, about 25% of human cancers exhibit defects of HR.^12^ Further exploitation in the defect of DNA repair mechanism offers a potential target for the anticancer drugs, as the prolonged existence of DNA double-strand breaks (DSBs) leads to cellular cytotoxicity.^13^ Therefore, developing studies assessed the potency of HR inhibitors as anticancer drugs. The target proteins of those HR inhibitors include ataxia-telangiectasia mutated (ATM), checkpoint kinase (CHK) 1 and 2, ataxia telangiectasia and Rad3-related protein (ATR), and Brca1.^12,14^ Another mechanism of DNA repair is base excision repair, which is facilitated by poly (ADP-ribose) polymerase (PARP).^15^

Curcumin, from turmeric (*Curcuma longa*), shows potency to act as a chemopreventive agent targeting genome instability and mutation (Table 1). Curcumin targets 3 major DNA damage repair pathways: non-homologous end joining (NHEJ), homologous recombination repair (HRR), and DNA damage checkpoint response (DDR).^16,17^ Curcumin suppresses NHEJ through inhibition of histone acetyltransferase activity and suppresses HR pathways by inhibiting *Brca1* expression.^17^ Curcumin also alleviates the activation of ATR-CHK1 signaling.

**Table 1:** Indonesian plants and/or their components that show chemopreventive activity targeting cancers’ genome instability and mutation

**Molecular target**	**Plant/compounds**	**Reference(s)**
CHK 1 (↓)	Curcumin	16
ATR (↓)	Curcumin	17
Histone acetyltransferase (↓)		
Brca1 (↓)		

CHK1: checkpoint kinase 1; ATR: ataxia telangiectasia and Rad3-related protein; Brca1: Breast cancer type 1 susceptibility protein. ↓: indicates down-regulation of a protein and/or pathway.

### Sustaining proliferative signaling

Cell proliferation regulators consist of inducers (growth factors, receptor tyrosine kinases/RTKs, transcription factors) and effectors. While the proliferation of normal cells is strictly regulated, cancer cells exhibit aberrant regulation of cell proliferation.^18^ Cancer cells may overexpress either the receptors or ligands of growth factors, such as androgen receptor (AR) in prostate cancer and estrogen receptor (ER) in breast cancer.^19,20^ RTKs are also commonly deregulated in the cancer cells, including epidermal growth factor receptor (EGFR), human epidermal growth factor receptor 2 (HER2), and Bcr-Abl.^21,22^ In addition, aberrant activation of transcription factors are also important for carcinogenesis. The overactivation of NF-κB plays an extensive role in carcinogenesis.^23^ The role of c-Myc as a proto-oncogene has been well-established and the overexpression of c-Myc is often associated with poor prognosis in the cancer patients.^24^ The effector of cell proliferation is cell cycle machinery, which consists of cyclins and cyclin-dependent kinases (CDKs) as the regulatory proteins. About 15%–40% of cancer shows amplification of *CCND1*, cyclin D1 gene.^25^ The overexpression of cyclin E associates with trastuzumab resistance in HER2-positive breast cancer patients.^26^

Various Indonesian medicinal plants show chemopreventive activity by inhibiting the proliferative signaling pathways in cancer cells (Table 2). Thymoquinone selectively inhibits the proliferation of prostate cancer cells by suppressing AR overactivation.^27^ Curcumin also inhibits AR expression in prostate cancer cells.^28^ The aqueous extract of *B. javanica* attenuates EGFR activity in liver cancer and human non-small-lung cancer cells; resulting in the inhibition of cancer proliferation.^29,30^ Ethanolic extract of *C. sappan* inhibits HER2 expression in breast cancer cells.^31^ Interestingly, curcumin shows an impressive activity against various RTKs, including EGFR and HER-2, in colon cancer cells.^32^

**Table 2:** Indonesian plants and/or their components that show chemopreventive activity targeting cancers’ proliferative signaling

**Molecular target**	**Plant/compounds**	**Reference(s)**
AR signaling (↓)	Thymoquinone Curcumin	27 28
EGFR (↓)	*Brucea javanica* (aqueous extract)	29,30
	Curcumin	32
HER2 (↓)	*C. sappan* (ethanol extract)	31
	Curcumin	32
NF-κB signaling (↓)	Bruceajavanone B	33
	Bruceine A	33
	Brusatol	34
	Panduratin A	35
c-Myc (↓)	Bruceantin	36
Cell cycle (CDKs/cyclins) (↓)	Panduratin A (cyclin D1, cyclin E; CDK 2, 4, 6)	37
	Brazilein (cyclin D)	38

AR: androgen receptor; EGFR: epidermal growth factor receptor; HER2: human epidermal growth factor receptor 2; CDK: cyclin-dependent kinase. ↓: indicates down-regulation of a protein and/or pathway.

Bruceajavanone B, bruceine A, and brusatol (from *B. javanica*) inhibit the activation of NF-κB in leukemia and pancreatic cancer.^33,34^ Panduratin A also shows its anticancer effect in lung cancer cells by inhibiting the activation of NF-κB.^35^ Bruceantin down-regulates c-Myc in multiple myeloma cells, leading to the inhibition of cell proliferation and induction of apoptosis.^36^ Panduratin A down-regulates multiple cell cycle regulatory proteins in prostate cancer cells, including cyclin D1, cyclin E, Cdk 2, Cdk 4, and Cdk 6.^37^ Brazilein, from *C. sappan*, down-regulates cyclin D1 and induces cell cycle arrest in G1 phase in MCF-7 breast cancer cells.^38^

#### Evading growth suppressor

Tumor suppressors have the eminent roles to inhibit the growth signaling cascades; however, their loss of function is frequently found in the cancer cells. Retinoblastoma protein (pRb) inactivates E2F, which is an important transcription factor for cell cycle progression, DNA replication, DNA damage repair, cell cycle checkpoint, and apoptosis.^39^ The p53 is another powerful tumor suppressor protein that up-regulates the gene expression of proteins involved in the cell cycle arrest, senescence, and apoptosis; serving as a barrier for the growth of cancer cells.^40^ Loss or mutation of p53 occurs in various cancers, including in 50% of non-small-cell lung cancer cases and skin cancer, above 70% of small-cell lung cancers, and almost 100% in high-grade serous carcinoma of the ovary.^41-43^

Phosphatase and tensin homolog (PTEN) is a phosphatase that serves as an important inhibitor for PI3K/Akt/mTOR pathway. Loss of PTEN occurs in various sporadic tumors.^44,45^ Other prominent growth suppressors are the Cdk inhibitors (Cdki), such as p16, p21, and p27. These proteins are activated by p53 and inhibit cell cycle progression from G1 to S phase.^46^

Various natural compounds exhibit the ability to restore the level and/or function of tumor growth suppressors (Table 3). Aqueous extract of *B. javanica* increases the level of p53 in breast cancer and cervical cancer cells.^47^ Thymoquinone increases the level of p53 in cervical cancer cells and up-regulates PTEN in both mRNA and protein level in doxorubicin resistance-breast cancer cells.^48,49^ Curcumin up-regulates the expression of multiple tumor growth suppressors, including p16/INK4a, p21/WAF1/CIP1, and p27/KIP1; as well as inhibits hyperphosphorylation of retinoblastoma (Rb) protein.^50^

**Table 3:** Indonesian plants and/or their components that show chemopreventive activity targeting cancers’ growth suppressors

**Molecular target**	**Plant/compounds**	**Reference(s)**
p53 (↑)	*Brucea javanica* (aqueous extract)	47
	Thymoquinone	48
PTEN (↑)	Thymoquinone	49
pRb(↑)	Curcumin	50
Cdki (↑)	Curcumin	50

PTEN: phosphatase and tensin homolog; pRb:  retinoblastoma protein; Cdki: cyclin-dependent kinase inhibitor. ↑: indicates up-regulation of a protein and/or pathway.

#### Enabling replicative immortality

By shortening of DNA telomere, normal cells would enter a phase called replicative senescence where they could not further proliferate. However, cancer cells escape from this event through various mechanisms; thus are able to proliferate continuously. Telomerase, a complex of enzymes repressed in normal cells, is activated in the cancer cells and preserves the telomere of cancer cells.^51^ Telomerase complex consists of various enzymes; one of the most important is telomerase reverse transcriptase (TERT; hTERT for human) that becomes the rate-limiting step in telomerase activity.^52^ Telomerase is found in about 85% to 90% of all malignant tumors and become an interesting target for the anticancer drugs.^53^ Several natural compounds show anticancer activity by targeting replicative immortality of cancer cells (Table 4). Methanolic extract of *C. sappan* was shown to inhibit telomerase activity in oral carcinoma and osteosarcoma cells.^54^ Curcumin also inhibits telomerase activity, which mediated by proteasome induced-degradation of hTERT.^55^

**Table 4:** Indonesian plants and/or their components that show chemopreventive activity targeting cancers’ replicative immortality

**Molecular target**	**Plant/compounds**	**Reference(s)**
Telomerase (↓)	*C. sappan* (methanol extract)	54
	Curcumin	55
hTERT (↓)	Curcumin	55

hTERT: human telomerase reverse transcriptase. ↓: indicates down-regulation of a protein and/or pathway.

#### Resisting cell death

Beside proliferate uncontrollably; cancer cells are also able to resist cell death (apoptosis). Apoptosis is initiated through two main pathways: intrinsic and extrinsic pathways. The intrinsic pathway is closely related with mitochondria permeabilization and regulated by the homeostasis of pro- and anti-apoptotic proteins, i.e. Bak, Bax, Bim (pro-apoptotic) or Bcl-2, Bcl-xL, Mcl-1 (anti-apoptotic).^56^ The extrinsic pathway starts with the activation of death receptors in the cell membrane, i.e. TNF-related apoptosis-inducing ligand (TRAIL) and Fas receptor.^57^ Those death receptors are activated by several ligands: TNF for TRAIL and Fas ligand (FasL) for Fas. The agonist of death receptor is promising to be developed as the anticancer agents.^58^

Various natural compounds are reported to overcome apoptosis resistance in cancer cells (Table 5). Brucein D, brusatol, brazilin, pinostrobin, and curcumin were reported to down-regulate Bcl-2.^34,59-62^ Curcumin suppresses important anti-apoptotic protein Mcl-1.^60^ Panduratin A up-regulates both Fas and TRAIL in prostate cancer cells.^37^

**Table 5:** Indonesian plants and/or their components that show chemopreventive activity targeting cancers’ cell death

**Molecular target**	**Plant/compounds**	**Reference(s)**
Bcl-2 (↓)	Brusatol	34
	Brucein D	59
	Curcumin	60
	Brazilin	61
	Pinostrobin	62
Mcl-1 (↓)	Curcumin	60
Fas (↑)	Panduratin A	37
TRAIL (↑)	Panduratin A	37

Bcl-2: B-cell lymphoma 2; Mcl-1: Induced myeloid leukemia cell differentiation protein; TRAIL: TNF-related apoptosis-inducing ligand. ↓: indicates down-regulation of a protein and/or pathway. ↑: indicates up-regulation of a protein and/or pathway.

#### Inducing angiogenesis

Cancer cells develop blood vessels independently from normal cellular physiology to support their need for nutrition and oxygen in a process called angiogenesis. During angiogenesis, cancer cells secrete various pro-angiogenic factors that stimulate endothelial cells to grow and produce various digestive enzymes.^63^ These factors include vascular endothelial growth factor (VEGF), platelet-derived growth factor (PDGF) and fibroblast growth factor 2 (FGF-2).

Without enough supply of oxygen from blood vessels, cancer cells suffer from a hypoxia condition. In this situation, hypoxia-inducible factor-1α (HIF-1α) is activated. And induces the expression of various genes that promote the mitogenic and migratory activities of endothelial cells.^64^ Hence, HIF-1α is an interesting molecule to target cancer angiogenesis.

Several natural compounds are reported to interfere angiogenesis in cancer cells (Table 6). Studies found that thymoquinone suppresses the level of VEGFR2; while ar-turmerone down-regulates VEGFR3.^65,66^ Curcumin down-regulates the transcriptional activity of HIF-1α under hypoxia, resulting in the suppression of VEGF level.^67^ Tetrahydrocurcumin decreases VEGF level in osteosarcoma cells and down-regulates HIF-1α, resulting in mesenchymal-epithelial transition/MET.^68^

**Table 6:** Indonesian plants and/or their components that show chemopreventive activity targeting cancers’ angiogenesis

**Molecular target**	**Plant/compounds**	**Reference(s)**
VEGFR (↓)	ar-turmerone	65
	thymoquinone	66
VEGF (↓)	Curcumin	67
HIF-1α (↓)	Curcumin	67
	Tetrahydrocurcumin	68

VEGFR: vascular endothelial growth factor receptor; VEGF: vascular endothelial growth factor; HIF-1α: hypoxia-inducible factor-1α. ↓: indicates down-regulation of a protein and/or pathway

#### Activating invasion and metastasis

Metastasis is responsible for >90% mortality of patients with solid tumors.^69^ In order to achieve metastasis, cancer cells produce various proteins that play important roles in cell-cell adhesion, cell-matrix adhesion, cellular migration, and epithelial-mesenchymal transition (EMT). E-cadherin is a key protein regulating cell-cell adhesion through the formation of the cell-cell junction. Loss of E-cadherin is found in the progression of tumor malignancy of most epithelial tumors.^70^ Down-regulation of E-cadherin occurs via various mechanisms: genetic or epigenetic mechanism, transcriptional suppression, proteolytic degradation, and modulation of several RTKs.^71^ Beta-catenin, while being an important protein for cell-cell adhesion, also serves as an oncogenic protein. Nuclear localization of β-catenin induces EMT resulting in metastasis.^72^ In cell-matrix adhesion, integrin plays a significant role to facilitate the interaction between cells and extracellular matrix (ECM).^73^ Other key proteins for invasion and metastasis are matrix metalloproteinases (MMPs). MMP-2 and MMP-9 degrade ECM components to facilitate cancer cells invasion and migration.^74^ Moreover, the overexpression of several MMPs could induce EMT.^75^

Numerous natural compounds exhibit inhibitory effect on invasion and metastasis (Table 7). *B. javanica* oil inhibits metastasis by up-regulating integrin.^76^ Thymoquinone reduces the expression of MMP-9, hence suppresses metastasis to multiple vital organs, including lungs, brain, and bone in the animal model of cancer.^66^ Thymoquinone down-regulates the expression of HER-2 and reduces the motility and migration of a highly metastatic pancreatic cancer cell line.^77^

**Table 7:** Indonesian plants and/or their components that show chemopreventive activity targeting cancers’ invasion and metastasis

**Molecular target**	**Plant/compounds**	**Reference(s)**
E-cadherin (↑)	Tetrahydrocurcumin	68
	Curcumin	85
MMP-2 (↓)	*C. sappan* (ethyl acetate fraction)	78
	Brazilein	79
	Panduratin A	35,82
	ar-turmerone	65
	Tetrahydrocurcumin	79
MMP-9 (↓)	*C. sappan* (ethyl acetate fraction)	78
	Brazilein	80
	Brazilin	81
	thymoquinone	66
	xanthorrhizol	83
	ar-turmerone	65,86
	Tetrahydrocurcumin	68
	Demethoxycurcumin	84
HER2 (↓)	*C. sappan* (ethyl acetate fraction)	78
	Thymoquinone	77
β-catenin (↓)	Tetrahydrocurcumin	68
Integrin (↓)	*Brucea javanica* (oil)	76

MMP-2: matrix metalloproteinase-2; MMP-9: matrix metalloproteinase-9; HER2: human epidermal growth factor receptor 2. ↓: indicates down-regulation of a protein and/or pathway; ↑: indicates up-regulation of a protein and/or pathway.

Ethyl acetate fraction of *C. sappan* decreases the protein level of MMP-2, MMP-9, and HER-2; thus inhibits cell migration in HER-2 overexpressed-breast cancer cells.^78^ Brazilein down-regulates MMP-2 and MMP-9.^79,80^ Whereas, brazilin decreases 12-O-tetradecanoylphorbol-13-acetate (TPA)-induced invasion in MCF-7 breast cancer cells through down-regulation of MMP-9 expression.^81^

Panduratin A suppresses the secretion and activation of MMP-2, resulting in the inhibition of endothelial cell migration, invasion, and morphogenesis in HUVEC cells and zebrafish embryo.^82^ In addition, the sub-toxic dose of panduratin A is sufficient to down-regulate MMP-2 in lung cancer cells.^35^ Xanthorrizol and demethoxycurcumin inhibits metastasis in mouse lung metastasis model and MDA-MB-231 breast cancer cells, respectively, through down-regulation of MMP-9.^83,84^ Curcumin increases E-cadherin.^85^ Tetrahydrocurcumin inhibits the migration and invasion of osteosarcoma cell line by increasing E-cadherin and suppressing MMP-2, MMP-9, and β-catenin.^68^ Finally, the sub-toxic dose of ar-turmerone inhibits metastasis through down-regulation of MMP-2 and MMP-9.^65,86^

#### Avoiding immune destruction

Cancer cells can avoid immune system through several ways, including (i) modification of immune regulatory cells, (ii) defective antigen presentation in the tumor cells, (iii) immune suppressive mediators, (iv) tolerance and immune deviation, and (v) induce apoptosis in immune cells.^87^ T regulatory (Treg) cell is a suppressor of the immune system. Tumor cells increase the suppressive activity of Treg and monoclonal antibody against Treg decreases tumor development.^88,89^ Natural killer (NK) cells also can be exploited to target cancer cells. NK cells were shown to control and/or eradicate some of the human hematopoietic tumors, as well as able to eliminate metastasizing cells and small tumor grafts.^90,91^ T cells, both helper and cytotoxic cells, were shown to have a positive impact on eliminating cancer. High expression of the Th1 cluster and CD8+ are positively correlated with prolonged disease-free survival in patients with colon cancer.^92^ At the molecular level, IL-12 shows various immunomodulatory activity, such as induces interferon-γ (IFN-γ) secretion and promotes the maturation of cytotoxic T cells.^93^

*Nigella sativa* and *Curcuma* genus show immunomodulatory activities against cancer cells (Table 8). The aqueous extract of *N. sativa* enhances the cytotoxicity of natural killer (NK) cells against YAC-1 tumor cells, indicating its potency as the stimulant for NK cells antitumor activity.^94^ Low concentration of thymoquinone increases the activation of CD8^+^ T cells and might beneficial for conditioning T cells *in vitro*, which will be used in T-cell therapy against cancer.^95^

**Table 8:** Indonesian plants and/or their components that show chemopreventive activity targeting cancers’ resistance to immune system

**Molecular target**	**Plant/compounds**	**Reference(s)**
NK cell activity(↑)	*N. sativa* (aqueous extract)	94
Th1 response (↑)	Curcumin	96
Treg lymphocytes (↓)	Curcumin	96
T-cell killer activity (↑)	Thymoquinone	95
	Curcumin	96
IL-12 (↑)	ar-turmerone	97

NK: natural killer; Th1: T helper cell type 1; Treg: regulatory, IL-12: interleukin 12. ↓: indicates down-regulation of a protein and/or pathway; ↑: indicates up-regulation of a protein and/or pathway.

Curcumin shows extensive activity against various types of T cells.^96^ Curcumin also enhances the response of Th1 (T helper cells) and the cytotoxicity of T killer cells. In addition, curcumin down-regulates Treg, a suppressor of the immune system. Ar-turmerone, another compound isolated from *Curcuma* genus, has been shown to increase the level of IL-12 in dendritic cells, which could be beneficial for the anticancer immunotherapy.^97^

#### Tumor-promoting inflammation

About 25% of tumor is closely related to chronic inflammation as chronic inflammation promotes tumor cell survival, proliferation, invasion, angiogenesis, metastasis, chemoresistance, and radioresistance.^98,99^ Moreover, chronic inflammation also may generate reactive oxygen species (ROS) and reactive nitrogen species that could induce the initiation and/or promotion of carcinogenesis.^100^ Therefore, tumor inflammation is a desirable target for a chemopreventive agent.

NF-κB serves as a transcription factor for various pro-inflammatory enzymes and cytokines.^101^ However, as NF-κB also closely related to the activation of immune system, NF-κB inhibitors should be designed carefully so that it does not impair the immune system.^102^

Cyclooxygenase-2 (COX-2) is a well-known enzyme responsible for inflammation events as it mediates the synthesis of pro-inflammatory molecule PGE_2_. Overexpression of COX-2 was found in various cancer tissues and selective inhibition of COX-2 might be beneficial for the prevention of cancer.^103-105^ Another important enzyme for inflammation is nitric oxide synthase (iNOS) that catalyzes the production of nitric oxide (NO), a potent pro-inflammatory mediator.^106^

Cytokines, such as tumor necrosis α (TNF-α), interleukin 1β (IL-1β), and interleukin 6 (IL-6), are also shown to correlate with tumor-promoting inflammation. TNF-α supports cancer initiation since it stimulates the production of genotoxic molecules, such as ROS and NO. TNF-α and IL-1β also develop a positive feedback loop with NF-κB, resulting in the sustained chronic inflammation in the tumor cells.^107^ IL-6, another type of cytokine, promotes apoptosis resistance in tumor cells during the inflammatory process.^108^

Various natural compounds exhibit anti-inflammatory activity that might beneficial to counteract tumor-promoting inflammation (Table 9). Panduratin A suppresses COX-2 expression level in colon cancer cells.^109^ Brazilin suppresses lipopolysaccharide-stimulated iNOS in RAW 264.7 macrophage cells; as well as inhibits the DNA binding activity of NF-κB.^110^ *B. javanica* oil has been shown to down-regulate the expression of COX-2 and p65, an active subunit of NF-κB.^111^

**Table 9:** Indonesian plants and/or their components that show chemopreventive activity targeting tumor-promoting inflammation

**Molecular target**	**Plant/compounds**	**Reference(s)**
COX-2 (↓)	*Brucea javanica* oil	111
	Panduratin A	109
	Thymoquinone	66,112
	Xanthorrhizol	113
	Curcumin	32
iNOS (↓)	Xanthorrhizol	113
NF-κB signaling (↓)	*Brucea javanica* oil	111
	Brazilin	110
	Thymoquinone	112
	Curcumin	114
TNF-α (↓)	*N. sativa* (aqueous extract)	94
	Curcumin	114
IL-6 (↓)	*N. sativa* (aqueous extract)	94
	Curcumin	114
IL-1b (↓)	Thymoquinone	112

COX-2: cyclooxygenase 2; iNOS: nitric oxide synthase; NF-κB: nuclear factor kappa-light-chain-enhancer of activated B cells; TNF-α: tumor necrosis factor α; IL-6: interleukin 6; IL-1b: interleukin 1b. ↓: indicates down-regulation of a protein and/or pathway.

The aqueous extract of *N. sativa* suppresses the secretion of IL-6 and TNF-α in primary macrophages cells.^94^ Thymoquinone decreases the expression level of COX-2 in pancreatic cancer and breast cancer cells.^66,112^ Furthermore, thymoquinone decreases the level of IL-1β, as well as inhibits the activation of NF-κB.^112^

Xanthorrizol inhibits the enzyme activity of COX-2 and iNOS in macrophage cells.^113^ Curcumin has been reported to suppress various inflammatory cytokines, including TNF-α, IL-1, IL-2, IL-5, IL-6, IL-8, IL-12, and IL-18. Curcumin is also a potent inhibitor of NF-κB transcription factor^114^ and decreases the expression level of COX-2.^32^

#### Deregulating cellular energetics

While glycolysis usually occurs in the normal cells under anaerobic condition, cancer cells are able to reprogram their energy metabolism mainly into aerobic glycolysis; the phenomenon that is known as the ‘Warburg effect’.^115^ Hypoxia is suspected as the basis of tumor cells metabolic reprogramming. When hypoxia occurs, the transcription factor HIF-1α is activated and induces the expression genes involving in glycolysis, including glucose transporter 1 (GLUT1), glucose transporter 3, hexokinase 2, pyruvate kinase 2, lactate dehydrogenase 5, and pyruvate dehydrogenase kinase 1 (PDK1).^116^ Therefore, HIF-1α serves as a key molecule for the anticancer drugs targeting cancer metabolism.

Cancer cells also suffer from a high level of ROS that is closely related to metabolism dysregulation in cancer cells. While a moderate level of ROS activates the expression of pro-survival genes, such as *HIF1A* and*GLUT1*, a high level of ROS in cancer cells trigger metabolism dysregulation and protein translation, resulting in the increase of ROS production.^117^ As excessive ROS level is toxic to cells, cancer cells upregulate various antioxidant systems, such as glutathione and thioredoxin antioxidant pathways.^118^ Developing studies reported that modulating ROS level is a potential strategy to eliminate various cancer cells. The combination of glycolysis inhibitor or glucose deprivation with inhibition of antioxidant systems, including glutathione and thioredoxin, exhibits synergistic anticancer effect breast and prostate cancer cells.^119^

Various compounds also exhibit anticancer activity targeting cancer cell energetic machinery (Table 10). A recent study showed that brusatol induces HIF-1α degradation in colon cancer.^120^ Furthermore, brusatol pre-treatment under hypoxia condition down-regulates the expression of HIF-1α downstream genes, including *GLUT1* and *PDK1*. Curcumin blocks glucose uptake in GLUT1 expressing cells up to 86%.^121^

**Table 10:** Indonesian plants and/or their components that show chemopreventive activity targeting deregulating cellular energetics induced by cancer cells

**Molecular target**	**Plant/compounds**	**Reference(s)**
HIF-1α (↓)	Brusatol	120
GLUT1(↓)	Curcumin	121
	Brusatol	120
PDK1 (↓)	Brusatol	120
ROS (↑)	Brucein D	122, 123
	Xanthorrhizol	124
	Curcumin	125

HIF-1α: hypoxia-inducible factor-1α; GLUT1: Glucose transporter 1; PDK1: pyruvate dehydrogenase kinase 1; ROS: reactive oxygen species. ↓: indicates down-regulation of a protein and/or pathway; ↑: indicates up-regulation of a protein and/or pathway.

Brucein D generates superoxide and exhibits cytotoxicity in pancreatic cancer cells, but not in non-tumorigenic cells.^122,123^ Xanthorrizol and curcumin also exhibits pro-oxidative activity. Xanthorrhizol elevates ROS level in oral squamous cell carcinoma cells and concurrent treatment with antioxidant partly reverses the cytotoxic activity of xanthorrhizol.^124^ Recent study showed that curcumin directly binds to various enzymes involving in ROS metabolic pathway; hence elevates ROS level in cancer cells and ultimately eliminates cancer cells.^125^

## Discussion

Indonesian plants and their bioactive components show promising activity as the anticancer and chemopreventive agents targeting the complex system of cancer. After a thorough investigation, we revealed the activity of Indonesian plants and their components against ten hallmarks of cancer (Tables 1-10). We could see that some constituents target more than one hallmark of cancer, even interfere with all hallmarks of cancer. Such ‘powerful’ compounds are curcumin (10 hallmarks), thymoquinone (7 hallmarks), and panduratin A (5 hallmarks). Despite their extensive targets on cancer cells, curcumin, thymoquinone, and panduratin A remain selective to eliminate cancer cells compared to the non-cancerous cells.^32^ These studies strengthen the urgency to develop natural compounds from Indonesian plants for clinical use in cancer therapy. However, curcumin and thymoquinone exert poor water solubility (<1.0 mg/mL), while the water solubility of panduratin A has not been well-established yet^126,127^; leading to a problem for drug formulation. Hence, scientists are concerting their effort to solve this problem, i.e. use curcumin and thymoquinone as the lead compounds to design the more water-soluble drugs or formulate these compounds with various techniques to increase their water solubility.

In their renowned paper about hallmarks of cancer, Hanahan and Weinberg acknowledge that combination chemotherapy (co-chemotherapy) is the key to effective cancer treatment. However, the co-chemotherapy strategy should be formulated on rational cancer targets by targeting multiple pathways or hallmarks of cancer.^3^ Targeting only one pathway or feature of cancer risks in cancer resistance and therefore, integrative and broad-spectrum co-chemotherapy serves as a potential strategy to combat cancer.^128^ Even though some of the plants or compounds do not act as a broad targeting-agent like curcumin, they are still promising to be developed as the anticancer and chemopreventive agents. *B. javanica* and its constituent exhibit their anticancer activities mostly via targeting cancer cells proliferative signaling (Table 2); whereas *C. sappan* and its constituents are potent anti-metastatic agents (Table 7). Meanwhile, *Curcuma* genus and *N. sativa* show promising activity against tumor inflammation (Table 9). Hence, further study could be conducted to determine their best combination as an integrative and broad-spectrum co-chemotherapy. To achieve that goal, the researchers should evaluate the synergistic anticancer effect of the combination of extract, fraction, or even pure compounds of various plants in an integrative experimental model of cancer.

## Conclusion

In summary, *Curcuma* genus, *B. javanica*, *B. pandurata*, *C. sappan*, and *N. sativa* show extensive anticancer and chemopreventive activities against various hallmarks of cancer. We recognize that only limited plants and compounds could be illustrated in this current article due to space limitation. Therefore, further experimental research and systematic reviews are quintessential to elucidate the anticancer properties of Indonesian plants, as well as develop them to be used clinically for cancer patients.

## Ethical Issues

Not applicable

## Conflict of Interest

There is no conflict of interest in this study
